# “*Always opening and never closing*”: How dialogical therapists understand and create reflective conversations in network meetings

**DOI:** 10.3389/fpsyg.2022.992785

**Published:** 2022-09-23

**Authors:** A. E. Sidis, A.R. Moore, J. Pickard, F. P. Deane

**Affiliations:** ^1^School of Psychology, University of Wollongong, Wollongong, NSW, Australia; ^2^School of Humanities and Social Inquiry, University of Wollongong, Wollongong, NSW, Australia

**Keywords:** reflecting teams, dialogical therapy, family therapy, Open Dialogue, interpretative phenomenological analysis

## Abstract

Tom Andersen’s reflecting team process, which allowed families to witness and respond to the talk of professionals during therapy sessions, has been described as revolutionary in the field of family therapy. Reflecting teams are prominent in a number of family therapy approaches, more recently in narrative and dialogical therapies. This way of working is considered more a philosophy than a technique, and has been received positively by both therapists and service users. This paper describes how dialogical therapists conceptualise the reflective process, how they work to engage families in reflective dialogues and how this supports change. We conducted semi-structured, reflective interviews with 12 dialogical therapists with between 2 and 20 years of experience. Interpretative Phenomenological analysis of transcribed interviews identified varying conceptualisations of the reflecting process and descriptions of therapist actions that support reflective talk among network members. We adopted a dialogical approach to interpretation of this data. In this sense, we did not aim to condense accounts into consensus but instead to describe variations and new ways of understanding dialogical reflecting team practices. Four themes were identified: Lived experience as expertise; Listening to the self and hearing others; Relational responsiveness and fostering connection; and Opening space for something new. We applied these themes to psychotherapy process literature both within family therapy literature and more broadly to understand more about how reflecting teams promote helpful and healing conversations in practice.

## Introduction

Family therapy brings together members of a person’s social network, and takes a systemic view in the formulation of problems. Despite extensive evidence of the efficacy of various forms of family therapy (Carr, 2018a,b) less is known about how these therapies achieve positive change (Carr, 2010, 2016). The introduction of the reflecting team by Tom Andersen (Andersen, 1987) has been described as revolutionary in the development of family therapy (Brownlee et al., 2009). Andersen was influenced by a social constructionist epistemology and the works of Gregory Bateson (Bateson et al., 1963) and Humberto Maturana (Maturana and Varela, 1980). These writings emphasised the construction of many unique realities based on perspectives and interactions with the environment. Maturana’s ‘multiverse’ evoked many possible meanings, and many perceived worlds.

In his seminal paper outlining his approach, Andersen (1987) details the Milan model of family therapy, which included a reflecting team who would observe the interview with the family behind a one-way screen. The clinician interviewing the family would meet with the team, discuss the problems of the family, and the clinician would return with their formulation to the family. Andersen’s experiment was to invite the reflecting team to trade places with the family, so that the family could listen to the conversation and reflections on what they had heard in the interview. Andersen and his colleagues felt that this would offer a more collaborative experience for families and allowed “direct access to the ideas of the team” (Biever and Gardner, 1995). The other effect that this had was to change the way clinicians spoke about the families, and how new information could be introduced to family members in such a way as to allow them to choose what aspects felt more relevant and important to them.

In Andersen’s reflecting team approach, families were invited to construct their own meaning through listening to varying perspectives from members of the team. Conversations between team members were based on observations of the family, tentatively offered speculation on how family members may be relating to the problem, and inner sensations or images related to the problem. The aim of these conversations was to open up possibilities for the family, and allow them to decide what fit best with their experience. Importantly the stance of “both/and” rather than “either/or” allowed for a diversity of perspectives both between and within team members (Andersen, 1987). The delivery of multiple perspectives and responses to a problem is considered integral to this approach, allowing clients to witness “doubt and ambiguity” (Haley, 2002 pp. 31) within a team. Andersen argued that helpful conversations were those in which different versions or perspectives of the problem could lead to a shift in the family system.

The structure of reflective conversations in which the team of clinicians would talk to each other, but be heard by the family (within the same room, or on one side of the two-way screen), is unique to this approach (Bacigalupe, 2002). This shift in position for family members, from observed to observing, is intended to promote the co-construction of meaning in relation to the problem and potentially allow clients to take a reflective position on the discussion. Following team reflections, family members are invited to speak about aspects of the conversation that caught their attention, or what they had been thinking of during this time. Families are encouraged to choose the direction of further exploration or discussion of possible solutions to the problem (Andersen, 1987; Memmott, 1998; Pender and Stinchfield, 2012). Reflecting teams are widely used by family therapists internationally, and there is growing enthusiasm for the practice in both family therapy (Willott et al., 2012) and in supervision and training (Biever and Gardner, 1995; James et al., 1996; Castles, 2011). Reflecting team practices have been described with deaf clients (Munro et al., 2008); those with intellectual disabilities (Anslow, 2014); people with gambling problems (Garrido-Ferńandez et al., 2011); people with opiate addiction (Garrido-Fernández et al., 2017); those with eating disorders (Russell and Arthur, 2000); people in war-torn (Charlés, 2010) and residential settings (Faddis and Cobb, 2016) and with young children (Fredman et al., 2007). Reflecting team sessions have been found to increase family connectedness (Browne et al., 2020) and hope among family members (Egeli et al., 2014; Armstrong et al., 2018; Allan et al., 2019). Dialogical approaches such as Open Dialogue have taken up a modification of reflecting team practices as a core component of the therapy process (Sutela, 2012). The dialogical perspective inherent in the reflecting conversations aims to attend to the many voices present in a meeting and several landmark naturalistic studies have shown reduction in long term disability and service use in early psychosis (Seikkula et al., 2003, 2011; Aaltonen et al., 2011; Bergström et al., 2017). Open Dialogue was found to be superior to treatment as usual for recovery and reduction in disability for adolescents with severe mental health concerns (Bergström et al., 2022). Qualitative studies of dialogical approaches including reflecting teams indicate that family members and clinicians alike value these open conversations (Sidis et al., 2020) and find them helpful (Flåm, 2009; Garrido-Fernández et al., 2011; Pender and Stinchfield, 2014; Allan et al., 2019).

A few studies have used conversation analysis of dialogical therapy to describe the way in which therapists encourage hope and positivity between family members (Williams and Auburn, 2016) downgrade authority to emphasise knowledge of family members (Ong et al., 2020) and make inferences to reflect their close listening (Schriver et al., 2019). Reviews of the reflecting team literature have been conducted (Pender and Stinchfield, 2012; Willott et al., 2012; Harris and Crossley, 2021) each espousing the need for further process research to aid in understanding how the reflecting team process achieves the shifts described. Despite the obvious association with reflective capacity which appears to be linked to efficacy in psychotherapy (Ekeblad et al., 2016; Bourke and Grenyer, 2017; Cologon et al., 2017), no studies to date have focused on how dialogical therapists encourage reflective conversations between family members. The current study aims to illuminate the variety of ways in which dialogical therapists understand, describe and encourage reflective conversations among family members. It also explores what these practices achieve in relation to the experiences of practitioners and participants in reflecting team meetings.

## Materials and methods

### Procedure

#### Participants

Purposive sampling was undertaken by inviting members of an Australian dialogical therapy interest group (sent information *via* email) and an international social media dialogical practice interest group (information posted to the site encouraged participants to contact the lead author).

Twelve dialogical practitioners from a variety of academic backgrounds participated in the study. One participant identified as a service user and practitioner. Eight participants were Australian, with two from Europe and two from the United States. Eight of the 12 participants identified as male and four as female with ages ranging from 30 to over 60 years. Participants practiced in various work contexts including community, outpatient, inpatient, and private practice. Experience in the Open Dialogue approach ranged from 2 to 5 years to greater than 20 years. See Table 1. Study methods were reviewed and approved by the local Human Research Ethics Committee (2021/064) prior to study commencement.

**Table 1:** Study participants.

Participant	Years of Open Dialogue experience	Workplace context	Discipline
P1	2–5	Community	Psychology
P2	2–5	Community	Nursing
P3	>20	Outpatient and private practice	Psychiatry
P4	2–5	Community	Social work
P5	2–5	Community and private practice	Psychology
P6	2–5	Community	Psychiatry
P7	11–15	Community	Psychiatry
P8	6–10	Inpatient	Nursing
P9	6–10	Private practice	Psychology
P10	6–10	Community	Psychology
P11	2–5	Community	Nursing
P12	16–20	Community	Family Therapy

#### Interviews

Twelve mental health professionals took part in semi-structured in-depth interviews. All interviews were conducted using zoom video conferencing software and were 90 min in duration. The first author who is a clinical psychologist conducted all interviews. Interview questions were developed *a priori* by the research team and included questions such as, “How do you think reflective talk emerges in your work with families?” and “What actions have you taken to support reflective processes or reflective talk among family members?” Although the interview focussed on the participants’ experience of reflecting teams and on how these therapists conceptualise and encourage reflecting talk among family members participants were encouraged to speak freely on aspects of practice that were relevant or important. Interviewees were asked to describe practice experiences alongside theoretical understandings of reflective processes in network meetings. Interviews were conducted from a social constructionist and dialogical perspective, in which the interview is understood as a setting for social discourse and the production of personal narratives (Tanggaard, 2009; Kvale and Brinkmann, 2015). In line with the critique of qualitative interview research described by Bøe and colleagues, a particular intention during the interviews was to attend to differences between participants, expressions of uncertainty and the variety (Bøe et al., 2021) of actions therapists may engage in as part of their therapy work.

#### Analysis

Transcripts were recorded and transcription was conducted by the first author. Given the intention of this study was to attend to both ideographic and across group patterns, an Interpretative Phenomenological methodology (Allan and Eatough, 2016; Smith, 2017, 2018) was applied to the recorded transcripts. The analysis was informed by Bakhtin’s dialogism (Bakhtin and Emerson, 1984) that recognises that meaning is created between participants and that each utterance is inherently polyphonic. We also considered the *qualitative fallacy* described by Bøe et al. (2021) in our analysis and attended to complexity and contradictions in the data and to participant uncertainty and hesitation evident in transcripts. The analysis included initial immersion in the data, with the first author conducting the interviews, reviewing transcripts and several close readings of all transcripts in full. Notes and annotations were made in the text, from which further reflections on divergent themes, along with individual participant’s experiences, were considered. As described by Smith and Shinebourne (2012), the hermeneutic circle method was used to relate participant’s experiences to broader themes using an explorative reflexive approach (Binder et al., 2012). Through an iterative process, themes were produced, however variation and contradictions to emerging themes were also considered. A dialogical approach to interpretation (Wells et al., 2020) was undertaken in which members of the research team with experience in various psychotherapy approaches and in linguistic discourse analysis met to interrogate these emerging connections from diverse perspectives. Finally, themes alongside idiographic conceptualisations and understandings of reflective processes were refined.

## Results

Participants’ descriptions and conceptualisations of the reflecting team process contained multiple perspectives on therapist actions and on the understanding of what reflecting teams achieve in the therapy context. While descriptions centred on the reflecting team process, many aspects were linked with general dialogical therapeutic principles. In line with the aims of this study, the focus of analysis stayed close to experiences in clinical practice or in Open Dialogue training and supervision.

Four interrelated themes were identified to capture both the way in which participants conceptualise reflective conversations in these family and social network meetings, and how these conversations are created:

1. Lived experience as expertise.
2. Listening to the self and hearing others.
3. Relational responsiveness and fostering connection.
4. Opening space for something new.

### Lived experience as expertise

Participants describing their practice and conceptualisation of reflecting teams spoke about a shift in how expertise and knowledge are held in reflecting team conversations. They described a genuine curiosity and positioned themselves as co-creators of the therapy talk. This invited family members to relinquish more traditional expectations regarding expertise. Contrasting with the expectation that service users may hold of mental health professionals, participant 9 describes not just a shifting of the notion of who is expert but also that expertise is not a requirement for problem resolution.

I think that, yeah, it empowers them and it helps them maybe renegotiate this notion of the expert. they say that yeah, what brings us here is that we wanted to hear the opinions of the experts. And just by talking on a more personal level, like sharing emotions or sharing your understanding, I think it makes it…it helps them understand that they are the experts and or that there is nothing to be expert about.

This requires not only a genuine intereste in the lived experience of family members but also a levelling of authority. Participant 11 noticed a shift in both expertise and power:

So, you know, that's a sort of, but the idea that it takes, that it critiques that expert… expert position and is saying ‘Well, we're kind of one of you too, we're having these inner thoughts and our doubts. And so it is a kind of democratizing of… of this gathering, this group – of trying to work out what's happening, and how can we, you know, or make a difference

In the statement above the participant links the challenging of the expert position to a collaborative effort to understand and learn about each other. This privileging of lived experience over positional expertise allows for dialogue without rank (Bakhtin and Emerson, 1984). They also include the sharing of inner thoughts and doubts, promoting the democratising of the space. The imagined group meeting in which all members hold power and agency to “make a difference” is joined together in their choice of the pronoun “we.”

Similarly, participant 1 describes both the elevating of lived experience and the tentativeness of their professional voice in the reflecting team process:

I think you give epistemic authority to the client, and the family, like the knowledge. So you ask things in a way that values their perspective rather than yours. And then similarly, when you offer yours, it's like what… what has been recommended, it's done in that way that is tentative… and that seeks feedback. is never stated as factual interpretation of their experience… is always offered as something that can be disagreed with.

Here the practitioner’s actions are linked with valuing the lived experience of family members along with an invitation to be an agent in the direction of therapy talk, or what is spoken about, and who can speak. Valuing each family member’s perspective and the expertise that they hold by virtue of lived experience invites an equal position and an opportunity to join as an active participant. This invitation to participate holds within it an openness to a different perspective, to attend to the talk on your own terms. Another practitioner (P2) speaks about how they describe the reflecting team process to family members:

We're going to turn to each other and… and look at each other and have this… reflect this in this way. To give you an opportunity to just listen to us without feeling like you're under the gun and you have to respond. And then after we do it, we're going to turn back to you and you get the last word.

Here an emphasis on reflecting team members speaking to each other, and family members being allowed to listen without perhaps the usual expectation to agree with clinicians in the meeting, conveys an epistemological shift. This process of team members speaking in front of the family but to each other can be likened to sitting in the back seat, rather than being a driver of a vehicle, where the participant is able to view the problem without having to respond to the discussion. Not being expected to respond either verbally nor in non-verbal expression as per social convention provides an opportunity to family members to hear and consider the problem and what is being said. Family members’ being handed the “last word” once again privileges their expertise and agency in the conversation.

### Listening to the self and hearing others

Dialogical therapy practitioners in this study also spoke about their own inner dialogues, attending to internal thoughts, sensations, emotions and images. This kind of listening was constructed as noticing one’s own inner self in a way that supported them to hear others and respond to them. Participant 10 describes this noticing of others as synchronised with noticing oneself:

I think for me, it's about having, like, genuine, like really authentic curiosity…like, my attention is drawn to this and it's almost like it's saying something to me, and I want to know more about it. Um, and so, so that's what's coming up for me in that process.

In this description of practice, curiosity is applied to both the family members’ verbal and bodily expressions as well as to the inner experience of the practitioner. Here, the practitioner uses their noticing of the self as a pathway to listening to the experience of others. The practitioner’s own internal experiences become eyes and ears. This movement from inner to outer worlds guides the actions in the meeting. Participant five speaks about the way in which this connection to self encourages this listening:

To connect with myself and to think right—How is my body? How is my mind? Am I present? Am I listening? What do I want to know more about? Why? What do I want to ask about that's been said? Yeah. To be. Yeah, to be oriented to my own experience.

Orienting to the way in which the act of attending to others ripples through our own inner experience seems akin to mindfulness and potentially opens the practitioner up to new understandings. Practitioners in this study considered this self-awareness as essential to being responsive to the needs of family members. Participant 9 reflects on this:

Yeah, I think that's the… It’s the freedom to share, but it's also the attentiveness to oneself, that is, like a prerequisite to be of best support to people, we talk about how we are in their presence or what they evoke in us

This self-awareness was also described by a number of practitioners who experienced reflecting teams during training and supervision. Being in the listening position during reflecting team talk also appeared to invite a similar connection between attending to the self while hearing others. Participant 9 describes the experience of being reflected on:

That you can understand yourself, be aware of yourself in some way because somebody has noticed something about you and then as you speak about it, you can you can hear yourself, see yourself as well as others getting to know you.

This noticing described above may relate to present moment changes in voice tone, or non-verbal expressions such that the person being reflected on may choose to connect what is spoken about to their own experience. One practitioner (P8) uses the auditory metaphor of an echo to describe their experience of being reflected on during reflecting team training:

When you think about a mirror, it's more like a one to one thing, but an echo doesn't sound like the real thing, but you can still make out what was said. So I think I heard… I heard myself through the other person.

To hear one’s own words and experiences spoken about by reflectors in this way offers an opportunity to experience this through the lens of another person’s life experiences and present moment responses. This implies a relational reflexivity (Burnham, 2018) in the way in which attending to both the self and others simultaneously provokes a deeper understanding for both reflectors and those experiencing a reflecting team.

### Relational responsiveness and fostering connection

Many of the participants in this study spoke about the way in which the reflecting team process engendered a sense of “being with” families (Shotter, 2005) and characterised this connection as essential to the process. This was created in a variety of ways including attending to emotional and bodily responses and staying present. Participant 9 described this:

I think you manage to connect in a way and through connection comes healing. And I think, I mean, through this more… making use of myself, in the sense of the emotions, and not so much the thoughts, I think, yeah, it allows them to…to be together at a more personal level.

Here healing and recovery is understood as a result of being together in a way that is described as personal. This is conveyed as a result of connecting to inner experiences. Other practitioners understood this connection in terms of physiological attunement (P6):

Our responses, you know, physiological responding to each other. Synchronization, that… you know, is actually that happening at a physiological level so that there is this kind of... ‘I'm connecting with what you're feeling’, and... and so, what they’re feeling actually gets somewhat amplified and noticeable and more comfortable.

A physiological attunement is understood here as allowing emotional responses to be seen (amplified) and acknowledged. This is described in the above as a connection with what others are feeling, which allows for both noticing and comforting others in the presence of difficult emotions. This acknowledgement of experience is associated with insight by another practitioner (P8):

And I think, at least to begin with, and therapeutic settings, it, the acknowledgement sits at the core of what caring is about. And I think that the acknowledgement is what allows other things to happen in terms of insight, but one thing is that the original speaker is.is sending out a signal. A noise. And then, for me, the first job of the clinician is to say that there's someone out here and you are being received.

The prioritisation of relationally responding is clearly articulated as the “first job,” and is portrayed as allowing for insight in reflecting team conversations. The acknowledgement of the family member’s experience is centred in the practice as what allows things to happen. The study participant’s choice to describe themselves as “someone out here” evokes a connection that assures the family member that they are not alone. This connection was also linked to staying present. Participant 4 describes their practice of present moment attending and responding to family members in a network meeting:

In this moment, you know, that I might think of all the things that you're going to ask me. But really, all I can do is respond to the things that you're saying to me right here right now. And that feels like a much more genuine thing to be doing, sticking to the present. As much as you want to talk about that thing that you didn't quite resolve in the last time you were all sitting together, actually this is a whole new piece of music.

The engagement of the auditory metaphor of music in this description not only serves to emphasise the changing nature of moment-to-moment interactions but also a sense of appreciation for the experience of being a listener in this context.

### Opening space for something new

This introduction of different voices, perspectives, and understandings in reflecting team talk has been described by Tom Andersen (Andersen, 1987) and others (Anderson and Jensen, 2007; Pender and Stinchfield, 2012; Shotter, 2015). Participants in this study described the moments that allow new information to emerge in network meetings. Participant 4 describes this curiosity and uncertainty as opening space for thinking differently:

You know, we use words around the space, always opening and never closing anything. It's recognizing the... the importance of... of things that are spoken together...what has been fed back to me is this idea, particularly from parents, of feeling like they've, they've really been heard, and that their experience has been felt, or that words have been shared about their experience that are different, that are making them think differently about what they had shared.

This participant makes a connection between feeling heard and thinking “differently” about their experience. The description of “always opening” can be understood as the practice of seeking to understand more, or to understand various perspectives and ways to experience the words being spoken. This is placed in opposition to “closing” which invokes a definitive, single truth. Similarly participant three discusses their perceptions on what closes conversations and what opens space for new things to emerge:

As reflecting team members we are discussing about our feelings, bodily sensations and nonverbal things that are in the room. When we are thinking about what kind of heaviness or pain or something there might be, um, well, to me, I feel it's opening space for something to come. Of course, sometimes, quite often people also talk about this metaphor of illness, that's so common. That's closing doors from understanding, when families start thinking about this illness in their kids, that is closing doors of wondering about what's going on.

The description above captures the uncertainty described in dialogical practice (Seikkula and Olson, 2003), which here is linked to wondering and learning more about an experiences. This is contrasted with the “closing doors” of certainty related to medicalisation of distress and mental experience. Another participant (P6) also reflected on this uncertainty in reflecting team conversations as not-knowing (Anderson and Goolishian, 1992) and the way in which this allows new ways of thinking about things to emerge:

Whereas if you missed the mark, in this loose kind of, you know, this kind of creates a position, I think, where the person can have that internal conversation with themselves again, and so they go, I don't think that's quite right. I think it is this, you know, you've got it wrong. But suddenly there is you know, something's happening, the way it's understood that can be brought in to the dialogue with the network too that can become new information or new understandings.

Here therapy participants are invited to disagree, and disagreement is represented as allowing for more information to be shared and different perspectives to be acknowledged. This process not only describes the co-development of new ideas but also the recognition that family (and reflecting team) members may learn things about each other that were previously unspoken.

## Discussion

Dialogical therapists participating in this study described a variety of practices and understandings in their psychotherapy work. The aim of this study was to understand more about how practitioners conceptualise reflective conversations and about what actions they take to encourage them. Our secondary aim was to make sense of the positive responses to reflecting team practice from both practitioners and family members (Naden et al., 2002; Fishel et al., 2010; Willott et al., 2012; Egeli et al., 2014; Sidis et al., 2020; Harris and Crossley, 2021) and reports of improved outcomes compared to standard treatments for both reflecting teams (Brownlee et al., 2009; Garrido-Ferńandez et al., 2011; Garrido-Fernández et al., 2017; Armstrong et al., 2018) and Open Dialogue (Seikkula et al., 2006, 2011; Gromer, 2012; Bergström et al., 2018, 2022). We explored transcripts from in-depth interviews with dialogical therapists using Interpretative Phenomenological Analysis (IPA). This method was chosen as a means to illuminate divergence as well as convergence in the data. We also embraced dialogical perspectives in considering pauses and hesitations during the interviews. Our findings link to theoretical and practice based understandings of dialogical therapy (Ong and Buus, 2021) and also provide detailed, nuanced perspectives of reflecting team practice and what this practice may achieve.

Our first theme, Lived Experience as Expertise, aimed to capture practitioners’ approach to both knowledge and power in the therapy setting. Laitila (2009) differentiated between horizontal and vertical expertise in family therapy by considering the intersection between the accumulated knowledge of an individual, including their lived experience (vertical) and the co-constructed knowledge achieved by utilising the resources of all present in a session (horizontal). Practitioners in this study described a respectful inquiry into the lives and experiences of family members along with tentative offerings of their own present moment experiences in response to hearing them. These actions were often noted to be in contrast to mainstream therapy practices in which therapists are often positioned as expert knowledge holders. Efforts to dismantle positional power in mental health settings are becoming more prominent among mental health consumer groups (Gee et al., 2015; Holmes and Papps, 2018) and alternative approaches which directly consider the operations of forms of power have been recently developed (Johnstone and Boyle, 2018). These efforts recognise a harm described as epistemic injustice (Leblanc and Kinsella, 2016; Carver et al., 2017; Crichton et al., 2017; Naldemirci et al., 2021), caused by mental health professionals who may medicalise distress and inadvertently silence knowledge that arises from lived experience of that distress. One participant described reflective practices as democratizing the clinical setting. This is achieved through an authentic recognition of the value of knowledge gained through personal experience of the problem.

Anderson’s descriptions of Collaborative therapy, which has been influenced by Andersen’s reflecting team ideas and in turn influenced dialogical approaches, includes two important ideas related to this theme. Anderson’s collaborative therapy was based on the understanding of therapy interactions as meaning-making linguistic systems. This approach encourages clinicians to embrace genuine curiosity and to ask questions from a position of “not-knowing” rather than from a model or method that seeks specific answers (Anderson and Goolishian, 1992). In this way therapy participants can be invited to make sense of their experience in a way that does not privilege one person’s voice over another’s. This theme also connects to Bakhtin’s conceptualisation of “expressing authentic human life” which could only be achieved in dialogue without rank (Bakhtin and Emerson, 1984). Bakhtin understood dialogical conversations as those in which one participant’s utterance was presented as a response in some way to another participant. This he contrasted with monological conversations in which one participant speaks with little consideration of the experiences of the other or from a single perspective. But Bakhtin also stressed that utterances are, in a deeper sense, always “dialogic” in that “to speak or write is always to reveal the influence of, refer to, or to take up in some way, what has been said/written before, and simultaneously to anticipate the responses of actual, potential or imagined readers/listeners” (White, 2003).

There are of course some important caveats to treating reflective practice as a heteroglossic and democratising force. Firstly, the democratisation can only be partial as there are professional and legal responsibilities always in the background as potential meanings or actions that may need to brought to the fore. Critiques of the way that discourse has been “democratised” and “conversationalised” across professional and bureaucratic spheres such as medicine, law, and education caution that sometimes all these changes mean is that the power goes “underground” (e.g., Fairclough, 1992; Maley et al., 2013). Based on therapists’ responses in this interview study, we do not see reflective practice within therapy as an example of the kind of subterranean control that has been documented elsewhere. Secondly, as well as democratising relations between therapists and clients, reflective practice is also likely, at least within the therapy session, to affect hierarchical relations between family members—between the parents in a family; between parents and offspring; between siblings of different ages, genders, abilities, and interests, etc. Although this point was not explicitly made by interview participants, it is an important one to follow up in future research. The expanded dialogism of reflective practice, in which even the professional’s views are routinely questioned, could create positive “wiggle room” (Erickson, 2001) for new capacity and authority to speak within a family. Of course, this may not be without unsettling effects.

The second theme from this analysis listening to the Self and Hearing Others, describes practitioners’ attending to their own inner dialogues and experiences during the therapy talk. This is understood as important in order to respond to others in the meeting in such a way that they might feel heard. This adoption of therapist reflexivity during therapy conversations is not unique to reflecting teams (Brown et al., 2016; Bourke and Grenyer, 2017; Cologon et al., 2017), however, in using the reflecting team process, dialogical therapists share these inner experiences with clients in a way they hope might be helpful to them. Dialogical therapists participating in this study understood their own responses to therapy talk to be essential to guiding the conversation, and to the process of reflecting team practice. These two activities of noticing the self and noticing others appear to occur simultaneously and be mutually influential. Burnham described this *relational reflexivity* in which people are invited to be curious about the inner experiences of others as a means by which therapeutic relationships might develop and helpful conversations can occur (Burnham, 2018). Similarly, narrative therapist Johnella Bird used the term *relational consciousness* in her work (Bird, 2004). For her, noticing responses and experiences relationally, denotes a shift away from a judgmental stance towards an acknowledgement of the relational environment we live in. For all participants in therapy conversations, this may lead to connecting to un-tapped resources. These practices can also be linked to the concept of the relational mind (Bateson, 1972) in which the mind is understood as a system in constant interaction with the world and with other minds. Since Bateson and related authors inform many therapeutic approaches, it is interesting to ask what is distinctive about having therapists give voice to their own experiences and hear each other speak about those experiences in the therapy session itself, along with clients. And how might this particular mode of talk foster a specific kind of relating that works for family therapy?

As a partial answer to these questions, we suggest that attending to one’s own inner dialogues and experiences as a practitioner may also be understood as an orientation to self-experience in relation to others. There was a close link in practitioners’ descriptions between the expressions of family members and what practitioners shared during reflecting team conversations. How dialogical therapists come to decide what should be shared in reflecting team conversations may be associated with what Shotter describes as *action guiding anticipations and understandings* (Shotter, 2015). Taking up the work of Bakhtin, Shotter suggests that as we learn to be in dialogue, we construct our utterances in anticipation of a response from others. In order to do this we must be attuned to others and express these utterances in ways that reflect a sense that we are with them. Reflective practice reimagines who these others are, and thus how we attune to them.

This prioritisation of attunement is also present in our third theme Relational Responsiveness and Fostering Connection. Participants in this study described engaging a deliberate focus on embodied attunement as part of their practice. This attunement was understood as supporting practitioners in their attempts to be responsive to the needs and experiences of family members and to the development of trust and alignment. This experience of shared and co-created meaning is associated with healing and trust (Seikkula and Trimble, 2005). The practices described by therapists in this study include not only shared meaning, but also a sense of appreciating and attending to other’s experiences. Anderson (2012) describes *relationally responsive practice* as a way of being and philosophical stance. Taking up Derrida’s notion of hospitality (Larner, 1994) she emphasises the importance of acknowledging that we are both guest and host in the lives of families who seek support. This she describes as “being courteous, sensitive to their uneasiness, and careful” (p.16) but also to view the stories clients present as a *gift*, of fragments that unfold as client and therapist reflect together. The musical metaphor employed by one participant suggests that any given session would be considered to have new form, harmony and expressions of emotion than a prior piece of music (therapeutic interaction) which may have been very different across all these dimensions/aspects.

Our final theme—Opening Space for Something New—links closely to dialogical practice and the associated concepts of uncertainty and polyphony. Study participants’ descriptions of *opening* conversations also described a therapist position of not-knowing, which allow for a conversation considering possible options to emerge. Tolerance of uncertainty, considered a key element of Open Dialogue (Olson et al., 2014) relates to not rushing to make decisions about treatment too early in the process of a meeting. These decisions are made collaboratively and carefully considered in the context of the family’s current situation. Dialogical therapists in this study did not see themselves as holding more knowledge than the family members and instead described a focus on relational knowledge that is constructed in the dialogue. Embracing uncertainty about where the conversation is going, or how to best respond to the family, appeared to support unexpected and yet relevant stories and resources to emerge.

Bakhtin used the musical metaphor *polyphony* to describe dialogical interactions as inclusive of independent and equally important voices (Bakhtin and Emerson, 1984). Dialogical practitioners do not attempt to produce consensus or a single agreed truth but are instead interested in varying perspectives on problems. This applies not only to hearing from each person in the meeting, but also in attending to different voices within individuals. Our participants described being open to “wondering” about these perspectives, which appeared to create new ideas. During reflecting team conversations, practitioners also described being able to share opposing views about what they heard, so that family members get a sense of multiple perspectives on a problem between people, and perhaps even within a person. This practice perhaps permits family members to disagree with each other and with therapists and to open up new ways of thinking about the problem.

The reflecting team process described by Tom Andersen has been widely adopted and adapted and remains an unusual innovation in psychotherapy. Drawing on ideas from social constructionism, Maturana’s multiverse and dialogical philosophy the practices of reflecting teams privilege multi-voiced perspectives, lived experience and embodied responsiveness more than a model or technique. Practitioners participating in this study conceptualised these aspects of practice as key to recovery and healing. Engaging in this way may encourage both mental health professionals and service users to connect with present moment inner experiences as they occur in the context of meeting with others.

Although evaluative research into Open Dialogue is still in its infancy, a number of longitudinal naturalistic studies have shown better outcomes for young people with psychosis who have participated in this approach (Seikkula et al., 2006; Bergström et al., 2017, 2018) compared to those provided standard treatments. Family Psychoeducation for early psychosis also appears to be one of the few psychological interventions shown to reduce relapse rates (Leff et al., 1990; Leff, 2000; Harvey and O’Hanlon, 2013; McFarlane, 2016). Perhaps some of this can be attributed to simply involving the network in the treatment. Little is known about process factors in family therapies for psychosis (Grácio et al., 2016) although family cohesion is suggested to moderate general levels of distress in family members and young people (Brown and Weisman de Mamani, 2018). Other process studies in family based interventions for psychosis have emphasised therapists’ listening to participants’ experience, a needs focussed approach and developing a collaborative alliance (Grácio et al., 2016), all of which would seem likely to be enhanced by reflective practice given our findings above especially around the theme of listening to self and hearing others. Studies inquiring about family member’s experiences with family based approaches for psychosis indicate the importance of being responsive to the particular concerns of the participants (Sundquist, 1999) and attending to participants stories to understand the experience of psychosis (Buksti et al., 2006). Therapist responsiveness in dialogical therapy for psychosis has also been associated with shifts in client’s agency and a co-construction of words, meanings, and consequent emotional responses (Avdi et al., 2015), which also resonates with how practitioners in our study described reflective practice as a way of inviting client agency.

The themes identified in this study appear to relate to practices which may be considered common factors in helpful therapies (Wampold, 2015) and shift the conceptualisation of psychotherapies from medical discourses to conversations that promote healing (Wampold, 2001). These healing practices have been described as an emotionally confiding relationship with the healer, a healing context or ritual and a way of understanding or making meaning of distress (Frank and Frank, 1991). Reflective teams may also support network members to enter into personal reflections about themselves and others, a practice which has been described from an individualist perspective as mentalisation (Fonagy and Target, 2006) or metacognition in the literature on psychosis and severe mental illness (Lysaker and Dimaggio, 2014; Dimaggio and Lysaker, 2015). Increasing reflective capacity is proposed as a common aim across various forms of psychotherapy (Goodman et al., 2016) and therapists who show greater capacity for reflection tend to produce better outcomes for their clients (Bourke and Grenyer, 2017; Katznelson et al., 2019). This study may provide some insight into the outcomes observed for psychosis.

Finally, we note that the practitioners participating in this study relayed practice descriptions that were closely linked to the theory and literature relating to dialogical therapies. As a needs adapted approach, the content of network meetings may vary significantly across families, and even between meetings. This has added complexity to the measurement of fidelity to the Open Dialogue approach (Waters et al., 2021). Insights from the current study provide a greater understanding of the *approaches* some practitioners use in reflecting teams and dialogical therapy more broadly. Perhaps interviewing practitioners about their practice may be another way of ascertaining fidelity to an approach such as this one.

### Study limitations

This study explored the practices of dialogical practitioners with a specific focus on reflecting teams. Our in-depth interviews with 12 dialogical therapists are not representative of the international community of practice that exists today but aimed to provide insights into reflective teams in practice. These interviews are also not representative of all dialogical reflecting team practices or experiences with this approach. We acknowledge we focussed here on a particular outcome of the practice, that is, reflective conversations, and how these are generated. This may have skewed our participants’ descriptions of the practice and we may have missed negative or unhelpful experiences. Care was taken to make the interview prompts relatively neutral in order to avoid positive or negatively valanced responses. As indicated by the results, participants’ did not provide any descriptions of negative or unhelpful experiences. This may in part be due to their affiliation and commitment to a therapeutic approach that has reflective processes at its core. Future research might also include interview prompts that more explicitly ask about negative experiences. We also note that the lead author on this paper has trained in and provided Open Dialogue for 6 years, which is likely to be influential in the analysis. Other authors on this project who contributed to the dialogical analysis include two clinical psychologists with expertise in cognitive therapies, parent based interventions and attachment based approaches and an academic with experience in using linguistic analysis the study of psychotherapeutic and other clinical discourse. Further broadening of this analysis to include other relevant voices may have added to our findings. While our analysis and discussion has opened potential avenues for considering how these practices support recovery, further research is required to fully understand how these practices promote change. A particularly welcome next step would be to explore the authentic talk that constitutes reflective practice *via* recording therapy sessions, and to compare how reflective practice is conceptualised in theory, as discussed in the present paper, with what practitioners and clients actually do and say in therapy.

## Data availability statement

The raw data supporting the conclusions of this article will be made available by the authors, without undue reservation.

## Ethics statement

The studies involving human participants were reviewed and approved by University of Wollongong Human Research Ethics Committee. The patients/participants provided their written informed consent to participate in this study.

## Author contributions

AS was responsible for conducting all interviews, data analysis, and manuscript preparation. AM, JP, and FD contributed to data analysis and provided extensive comments on the manuscript in preparation for submission. All authors contributed to the article and approved the submitted version.

## Funding

This study was partially supported by an Australian Government Research Scholarship.

## Conflict of interest

The authors declare that the research was conducted in the absence of any commercial or financial relationships that could be construed as a potential conflict of interest.

## Publisher’s note

All claims expressed in this article are solely those of the authors and do not necessarily represent those of their affiliated organizations, or those of the publisher, the editors and the reviewers. Any product that may be evaluated in this article, or claim that may be made by its manufacturer, is not guaranteed or endorsed by the publisher.
